# Microbial sensing through the non-canonical inflammasome modulates airway type 2 immunity

**DOI:** 10.3389/fimmu.2026.1784561

**Published:** 2026-03-16

**Authors:** Olivier Bernard, Sheyla Yamato, Oluwaferanmi Bello, Raquel Alvarez, Carter J. Supple, Louis Z. Sharp, Jahanvi Kumar, Maya E. Kotas, Erin D. Gordon

**Affiliations:** Division of Pulmonary, Critical Care, Allergy and Sleep, Department of Medicine, University of California, San Francisco, San Francisco, CA, United States

**Keywords:** IL-33, lipopolysaccharides (LPS), non-canonical inflammasome, pyroptosis, type 2 immunity

## Abstract

**Introduction:**

Airway epithelial cells serve as critical sensors of both microbes and allergens, orchestrating immune responses through damage-associated molecular patterns including IL-33. Common aeroallergens induce type 2 inflammation through protease activity and pore-forming mechanisms that trigger epithelial IL-33 secretion and MAPK signaling. While microbial pattern receptors such as caspase-4 (which detects intracellular LPS) similarly generate membrane pores via the non-canonical inflammasome, it remains unknown whether these receptors can engage the same downstream epithelial IL-33 release and MAPK activation pathways.

**Methods:**

Using human airway epithelial cell models, we examined caspase-4-dependent pyroptotic signaling downstream of intracellular LPS, including gasdermin D cleavage, IL-33 release, and MAPK-dependent transcriptional responses. We assessed the modulatory effect of protease allergen co-exposure on LPS-induced pyroptosis and interrogated the role of Orai1-mediated calcium signaling *in vitro*. In a mouse model of protease allergen challenge, we evaluated innate type 2 immune responses following genetic deletion of caspase-4 (formerly caspase-11). LPS preparations from multiple bacterial species were tested for capacity to engage the non-canonical inflammasome in epithelial cells, and publicly available human asthma datasets were analyzed for airway expression of caspase-4 and gasdermin D.

**Results:**

Intracellular LPS activated caspase-4-dependent pyroptotic signaling, resulting in gasdermin D cleavage, IL-33 release, and MAPK-dependent transcriptional responses. Protease allergen exposure enhanced LPS-induced pyroptotic responses through Orai1-mediated calcium signaling in vitro. Genetic deletion of caspase-4 attenuated innate type 2 immune responses in the mouse protease allergen challenge model. LPS preparations from different bacterial species demonstrated variable capacity to engage the non-canonical inflammasome. Analysis of human asthma datasets revealed increased airway expression of both caspase-4 and gasdermin D in asthmatic patients relative to healthy controls.

**Discussion:**

These findings identify the epithelial non-canonical inflammasome as a pathway capable of linking microbial pattern recognition to IL-33-dependent type 2 responses. This work establishes a mechanistic framework for understanding how bacterial sensing machinery may intersect with allergic inflammation during pathophysiological conditions, and suggests that caspase-4 signaling could represent a therapeutic target in asthma.

## Introduction

Most cases of asthma are driven by type 2 inflammation, triggered or aggravated by environmental exposures (1–5). While allergens are the best studied environmental triggers, airway microbes also influence asthma development and severity (5). The exact relationship between asthma and the airway microbiome remains unclear, however, as epidemiologic studies show both positive and negative associations depending on exposure timing and bacterial species (6). For instance, while early-life exposure to diverse microbial flora may protect against asthma development (7), airway colonization by specific bacteria—including *Haemophilus, Neisseria, Fusobacteria*, and *Klebsiella*—correlate with asthma prevalence and severity in adults (8, 9).

Epithelial sensing and release of cytokines such as IL-33, IL-25, and TSLP is essential to the development and propagation of type 2 immune responses at mucosal barriers, acting through both innate and adaptive mechanisms (10, 11). These cytokines activate innate myeloid effectors like eosinophils, mast cells, and macrophages (4), stimulate innate lymphocytes including type 2 innate lymphoid cells (ILC2) to produce IL-5 and IL-13 to recruit eosinophils and initiate parenchymal remodeling (12), and induce differentiation of T helper type 2 (T_H_2) cells from naïve T cells (13). However, the mechanisms mediating allergen sensing remain incompletely understood. Many common allergens contain proteases that initiate type 2 immunity through cleavage of epithelial sensors such as proteinase-activated receptor 2 (Par2), inducing calcium signaling (14–17). Others activate the endogenous pore forming protein gasdermin D, leading to interleukin (IL-33) secretion (18). Some fungal allergens contain pore-forming toxins that directly permeabilize epithelial membranes, releasing IL-33 and initiating MAPK transcriptional signaling (19), while pollen-associated lipid mediators can directly disrupt epithelial barrier function, also leading to MAPK signaling (20, 21). These mechanisms converge on epithelial compromise, IL-33 secretion, and MAPK signaling to promote type 2 immunity. Whether epithelial microbial sensing synergizes with allergens to promote type 2 activation remains unclear.

Lipopolysaccharide (LPS), a cell wall component of gram-negative bacteria that is ubiquitous in environmental dust, may represent one mechanism linking the airway microbiome to type 2 immunity. LPS is detected by two mammalian sensors: Toll-like receptor 4 (TLR4) at the cell surface and caspase-4 intracellularly (22–24). The lipid A domain of LPS, which is recognized by both receptors, consists of phosphorylated and acylated glucosamine units whose specific structure varies widely among bacterial species, and this variation can determine the degree of activation (25–31).

Caspase-4 signaling differs fundamentally from that of TLR4. Upon binding LPS via its caspase activation and recruitment (CARD) domain, caspase-4 cleaves gasdermin D (GSDMD), releasing the GSDMD N-terminal domain which then oligomerizes and inserts into the plasma membrane to form pores (23, 32–35). This initiates pyroptotic cell death and secretion of IL-1 family cytokines, IL-1α, IL-1β and IL-18 (33). This caspase-4-dependent LPS-sensing pathway is referred to as the “non-canonical inflammasome, ” distinct from the canonical inflammasome wherein pattern recognition receptors (PRR) such as NLRP3 detect diverse stimuli and recruit caspase-1 for GSDMD activation (35). The non-canonical pathway thus provides a specialized mechanism for detecting intracellular LPS that complements other microbial sensing mechanisms.

Numerous studies link LPS-sensing to type 2 immunity, though the nature of the relationship remains unclear. In animal models, low doses of endotoxin enhance type 2 immunity via TLR4, while high doses suppress it (36, 37), and IL-33 secretion is partly TLR4 dependent (38, 39). Caspase-4 deficient mice are also resistant to ovalbumin-induced adaptive type 2 immunity (40), though the mechanism and whether this extends to innate type 2 lymphocytes is unclear.

IL-33 is an IL-1 family cytokine that initiates type 2 immunity (41–43). Like other IL-1 family members, IL-33 lacks a signal sequence and requires non-classical secretion. However, unlike IL-1β and IL-18, full length IL-33 is biologically active without proteolytic cleavage (44). Like IL-1α, full length IL-33 contains a nuclear localization signal (NLS) and histone binding domain (45), constraining most IL-33 to the nucleus under homeostatic conditions. An alternatively spliced transcript lacking exon 3–4 encodes a cytoplasmic IL-33 variant without an NLS that is readily secreted (46, 47). This variant is constitutively expressed in the lung epithelium from an alternative promoter, is induced by the inflammatory transcription factors AP-1 and AP-2 (48), and its expression correlates with asthma and chronic obstructive pulmonary disease in human studies (46, 47). However, the signaling mechanisms controlling airway epithelial IL-33 expression and secretion remain poorly defined.

Given the clinical association between asthma and airway microbial colonization, the role of LPS in modulating type 2 immunity, and the importance of caspase-4 in sensing intracellular LPS and secreting IL-1 family cytokines during pathophysiological conditions, we hypothesized that caspase-4 regulates type 2 immunity via epithelial injury and pore formation, leading to IL-33 gene expression and secretion. Here we show that LPS-induced caspase-4 activation triggers pyroptosis, MAPK signaling and IL-33 expression and secretion in airway epithelial cells. The protease allergen papain augments caspase-4-dependent pyroptosis and IL-33 secretion via store-operated calcium channels, and caspase-4 deficient mice exhibit reduced lung IL-33 secretion and innate type 2 responses. Pathway activation intensity varies by bacterial species. Finally, asthmatic patients have increased expression caspase-4 and GSDMD.

## Results

### Intracellular LPS induces epithelial pyroptosis and secretion of cytoplasmic IL-33

The human airway epithelium is where environmental aeroallergens are first encountered, is a site of constitutive IL-33 expression (46, 49), and unlike the more distal airway, is colonized by microbes (50). We hypothesized that airway epithelial cells respond to microbial products by activating the inflammasome and undergoing pyroptosis, a process first demonstrated in macrophages. *CASP4, CASP1* and *GSDMD* were similarly expressed in human primary alveolar macrophages, human primary airway epithelial cells cultured at air liquid interface and the BEAS-2B human airway epithelial cell line (**Supplementary Figure 1A**), indicating that IL-33-expressing airway epithelial cells also express the genes required for non-canonical inflammasome assembly.

**Figure 1:**
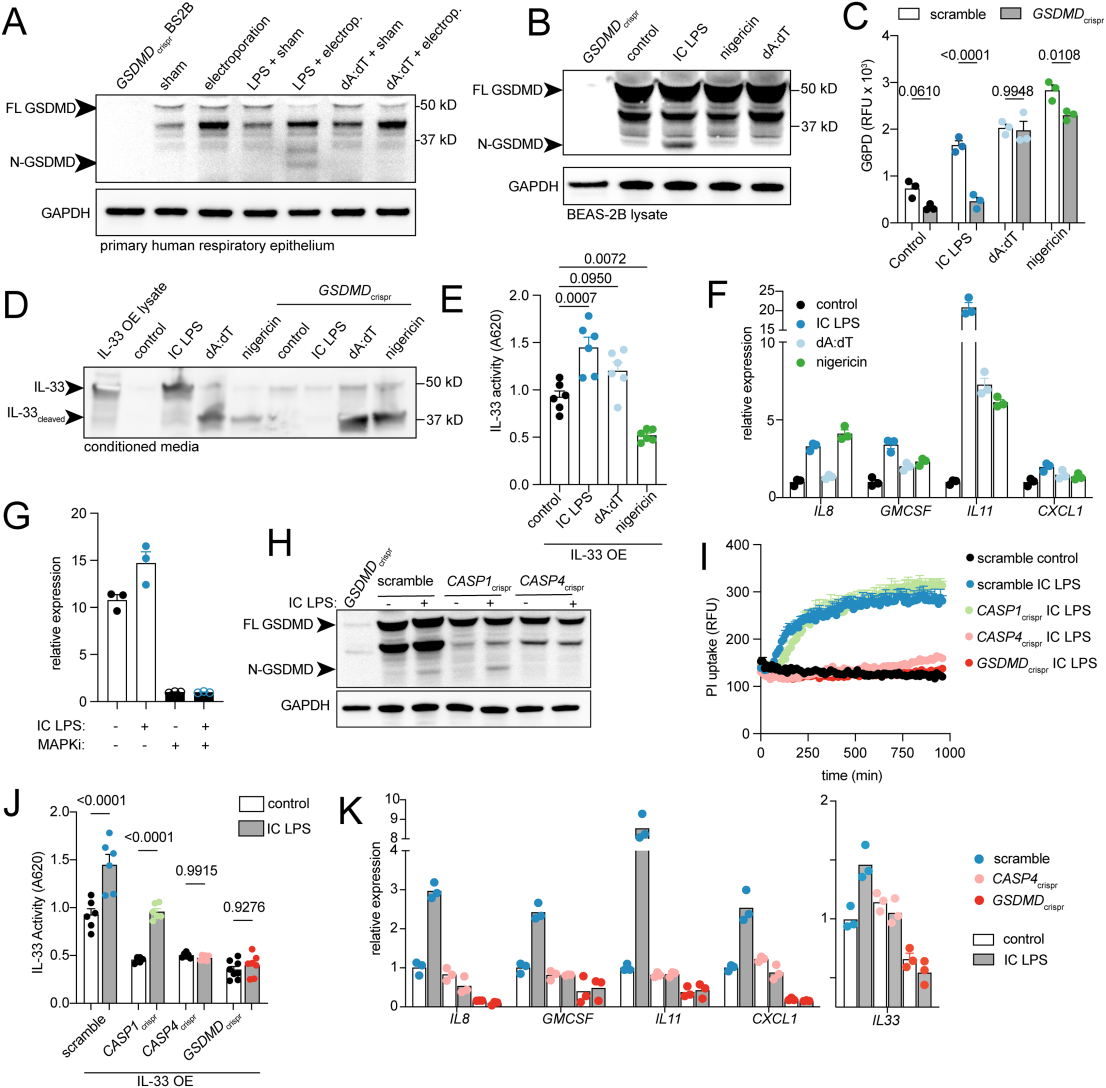
Non-canonical inflammasome-dependent pyroptosis and IL-33 secretion in airway epithelial cells. **(A)** Western blot from lysates of primary human airway epithelial cells cultured for 16 hours following electroporation of LPS or dA:dT (or sham). **(B)** Western blot of GSDMD and GAPDH from lysates of BEAS-2B cells treated with IC LPS and dA:dT for 3 hours or nigericin for 1 hour. **(C)** Glucose 6-phosphade dehydrogenase (G6PD) activity measured in media from BEAS-2B cells treated with IC LPS, dA:dT, or nigericin for 16 hours. **(D)** Western blot of IL-33 in concentrated supernatant from IL-33 OE BEAS-2B or *GSDMD_crispr_* BEAS-2B cells. **(E)** IL-33 activity measured by secreted embryonic alkaline phosphatase from HEK-Blue-IL33 cells treated with media from IL-33 OE BEAS-2B stimulated for 16 hours with IC LPS. **(F)** Gene expression for mitogen activated protein kinase (MAPK) target genes in BEAS-2B cells treated with IC LPS or dA:dT and nigericin for 4 hours. **(G)** IL33 gene expression in BEAS-2B cells treated with IC LPS or MAPK inhibitor (MAPKi, trametinib) for 4 hours. **(H)** Western blot of GSDMD and GAPDH from cellular lysates from CRISPR- BEAS-2B cell lines treated with IC LPS for 3 hours. **(I)** Propidium iodide (PI) cell uptake over time in CRISPR- BEAS-2B cell lines with indicated knockdowns. **(J)** IL-33 activity measured by secreted embryonic alkaline phosphatase from HEK-Blue-IL33 cells treated with media from IL33 OE CRISPR- BEAS-2B stimulated for 16 hours with IC LPS. **(K)** Gene expression for MAPK target genes and *IL33* from CRISPR-BEAS-2B stimulated for 4 hours with IC LPS. Data points reflect biological replicates. Error bars represent SEM. p values represent ordinary ANOVA with Sidak correction **(C, J)** or Tukey correction **(E)**.

Next, we sought to determine if airway epithelial cells undergo canonical (caspase-1 dependent) or non-canonical (caspase-4 dependent) inflammasome mediated pyroptosis, and whether this leads to secretion of IL-33. In macrophages, nigericin and intracellular dA:dT activate the canonical inflammasome via caspase-1, while intracellular (IC) LPS activates the non-canonical inflammasome via caspase-4; both pathways cleave GSDMD inducing pyroptosis and IL-1 release. To selectively activate the canonical or non-canonical inflammasome, we electroporated LPS or dA:dT into primary human airway epithelial cells. GSDMD cleavage occurred with IC LPS but not dA:dT (**Figure 1A**). To further dissect the mechanisms of inflammasome activation, we then turned to the genetically tractable BEAS-2B airway epithelial cell line. Like primary respiratory epithelial cells, these cells also showed GSDMD cleavage only with IC LPS, but not canonical inflammasome activators (**Figure 1B**). All stimuli—nigericin, dA:dT, and IC LPS—induced cell death, evidenced by uptake of propidium iodide (PI) (**Supplementary Figure 1E**) and release of glucose-6-phosphate dehydrogenase (G6PD) (**Figure 1C**). However, *GSDMD* disruption rescued IC LPS-induced death but not nigericin or dA:dT-induced death (**Figure 1C**; **Supplementary Figure 1G**). Conversely, caspase 3/7 activity increased with nigericin or dA:dT treatment but not IC LPS (**Supplementary Figure 1F**), indicating that airway epithelial cells undergo apoptosis in response to dA:dT and nigericin, but pyroptosis in response to IC LPS.

Since other IL-1 family members are secreted during inflammasome activation, we asked whether IL-33 could be secreted from cytosolic pools during airway epithelial pyroptosis. Most IL-33 protein is found in the nucleus under basal conditions, but cytosolic pools can be generated by alternative splicing or stress-induced nuclear translocation—both of which have been associated with disease pathophysiology. (18, 46, 47, 51). Reasoning that pyroptosis would most likely facilitate secretion from cytosolic pools, we engineered cells overexpressing either full-length nuclear IL-33 (“IL-33_NLS_ OE”) or the cytoplasmic variant of IL-33 (“IL-33 OE”) (46), both tagged with green fluorescent protein (GFP). Following IC LPS treatment, we detected IL-33 in conditioned media only from cytoplasmic IL-33-expressing cells (“IL-33 OE”) (**Figure 1D**) and not nuclear IL-33-expressing cells (“IL-33_NLS_ OE”) (**Supplementary Figure 1C**). After dA:dT or nigericin, an IL-33 immunoreactive band appeared but with substantially reduced molecular weight, suggesting proteolytic fragmentation (**Figure 1D**). To determine whether these various IL-33 reactive species have biological activity, we used a reporter cell line expressing *IL1RL1* (the IL-33 receptor) and an NF-KB/AP-1 inducible SEAP reporter. Biologically active IL-33 was detected in supernatants from cytoplasmic IL-33-expressing cells activated with IC LPS, but not with nigericin or dA:dT (**Figure 1E**). Supernatants from IL-33_NLS_ OE cells showed no biological IL-33 activity following canonical or non-canonical inflammasome activation (**Supplementary Figure 1D**), though an IL-33 cleavage product was again observed in dA:dT- and nigericin-treated cells, consistent with nuclear fragmentation during apoptosis. Thus, non-canonical inflammasome activation by intracellular LPS is sufficient to enable the secretion of cytoplasmic IL-33.

Recent studies show that epithelial membrane disruption by pore-forming toxins induces a MAPK-dependent transcriptional signature required for adaptive type 2 responses (19). We investigated whether endogenous pore formation from canonical or non-canonical inflammasome activation could similarly induce this pathway. Transcripts from the MAPK signaling cascade were robustly induced by IC LPS and less so by IC dA:dT and nigericin (**Figure 1F**). As with exogenous pore-forming toxins, induction was inhibited by the MAPK inhibitor trametinib (**Supplementary Figure 2C**). Moreover, though human airway epithelial cells constitutively express high levels of IL-33, we found that endogenous *IL33* transcripts were further induced in by IC LPS, and both baseline and induced *IL33* transcription were blocked by trametinib (**Figure 1G**). Together, these data establish that intracellular LPS activates a caspase-4 and gasdermin D-dependent pathway in airway epithelial cells that results in pyroptotic cell death, secretion of cytoplasmic IL-33, and MAPK-driven inflammatory gene expression.

### LPS-induced airway epithelial pyroptosis depends on caspase-4 and gasdermin D

To define requirements for inflammasome-dependent pyroptosis and IL-33 secretion in respiratory epithelium, we used CRISPR-Cas9 to disrupt caspase-1 and caspase-4 expression (**Supplementary Figure 1B**). IC LPS-induced GSDMD cleavage remained detectable in *CASP1*-deficient cells but was abolished in *CASP4*-deficient cells (**Figure 1H**). Both PI uptake and G6PD release following IC LPS were rescued in *CASP4*_crispr_ and *GSDMD*_crispr_ cells, while *CASP1* disruption partially reduced G6PD release without affecting PI uptake (**Figure 1I**; **Supplementary Figure 1H**). IC LPS-induced bioactive IL-33 secretion was also *CASP4* and *GSDMD-*dependent, with partial reduction in *CASP1*_crispr_ cells (**Figure 1J**, **Supplementary Figure 1I**). Finally, IC LPS-induced MAPK gene expression, including *IL33*, was suppressed in *CASP4*- and *GSDMD*-deficient cells (**Figure 1K**). Thus, IC LPS-induced cell death, bioactive IL-33 release, and epithelial inflammatory transcription are *CASP4-* and *GSDMD*-dependent.

### Epithelial pyroptosis and IL-33 secretion are modulated by papain and intracellular calcium

**Figure 2:**
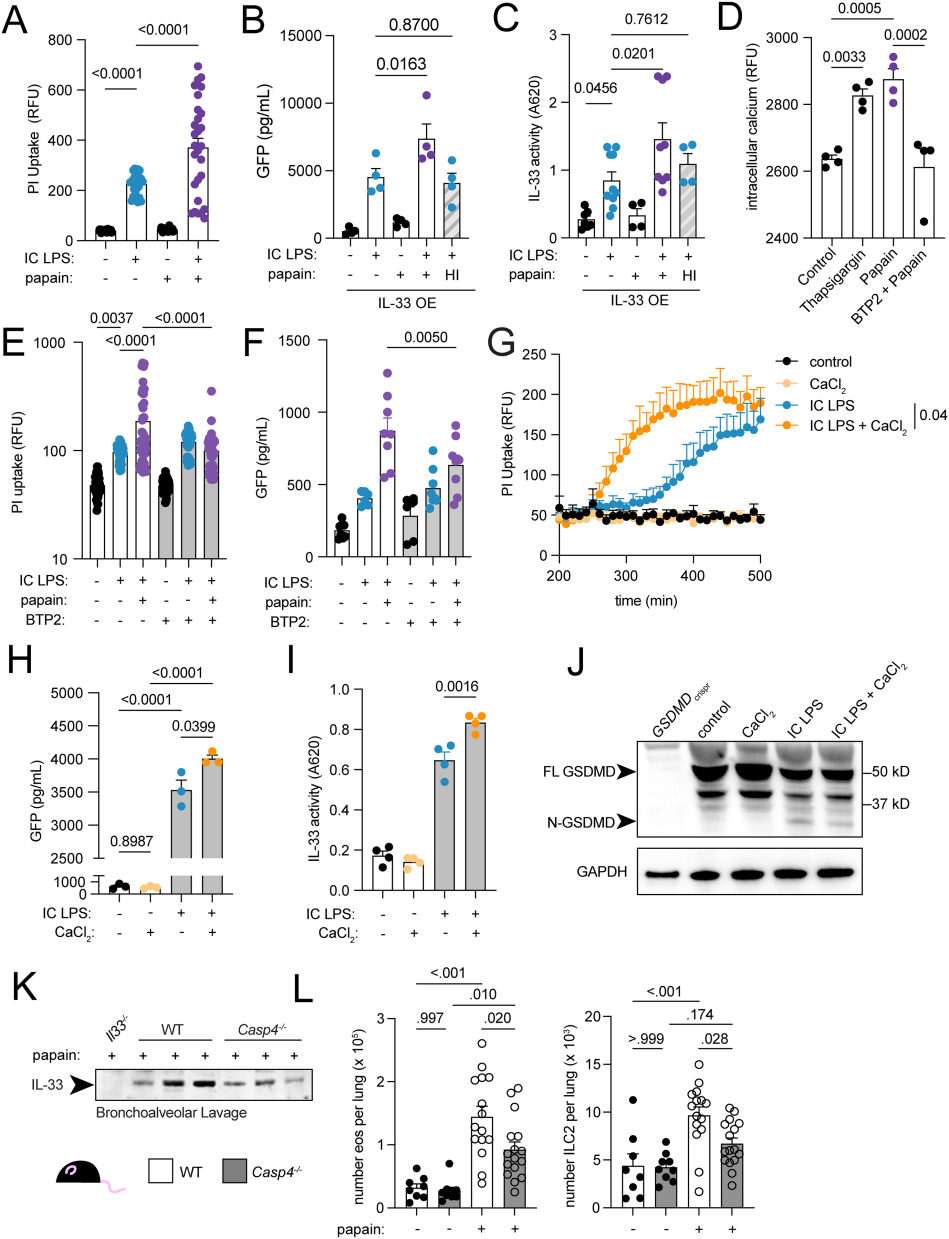
Caspase4-dependent epithelial pyroptosis and IL-33 secretion are modulated by papain via intracellular calcium. **(A)** Propidium iodide (PI) uptake at 6 hours in cells treated with IC LPS and papain. **(B)** GFP ELISA on conditioned media from IL-33 OE BEAS-2B cells treated with IC LPS and papain or heat inactivated (HI) papain for 16 hours. **(C)** IL-33 activity measured by secreted embryonic alkaline phosphatase from HEK-Blue-IL33 cells treated with media from IL-33OE BEAS-2B cells treated with IC LPS, papain and HI papain for 16 hours. **(D)** Intracellular calcium measured in BEAS-2B cells following thapsigargin, papain, or papain and BTP2. **(E)** PI uptake at 6 hours in BEAS-2B cells treated with IC LPS, papain and BTP2. **(F)** GFP ELISA on conditioned media from IL-33 OE BEAS-2B cells treated with IC LPS, papain and BTP2 for 16 hours. **(G)** PI uptake in BEAS-2B cells lines treated with IC LPS and calcium chloride. **(H)** GFP ELISA on conditioned media from IL33OE BEAS-2B cells treated with IC LPS and calcium chloride 16 hours. **(I)** IL-33 activity measured via HEK-Blue-IL33 cells in media from IL-33OE BEAS-2B cells treated with IC LPS and calcium chloride for 16 hours. **(J)** Western blot of GSDMD and GAPDH from cellular lysates from BEAS-2B cells treated with IC LPS and calcium chloride for 3 hours. P value represents two-way ANOVA with Tukey correction. **(K)** Western blot of BAL showing IL-33 protein in wildtype mice treated with 3 days of intratracheal PBS or papain. (**L**) Eosinophil and ILC2 counts from lungs of wildtype or *Casp4*^-/-^ mice treated with 3 days of intratracheal PBS or papain. Data points reflect biological replicates. Error bars indicate SEM. P values indicated for ordinary one-way ANOVA with Tukey correction.

We hypothesized that epithelial sensing of the airway microbiome and of allergens would synergize to promote IL-33 release and type 2 immunity. Because papain is a widely-used protease allergen that provokes IL-33 release in mouse models, we tested whether papain interacts with non-canonical inflammasome activation in airway epithelial cells. Papain alone did not induce cell death or IL-33 release from human airway epithelial cells. However, papain enhanced PI uptake and cytoplasmic IL-33 secretion in IC LPS-treated cells (**Figures 2A–C**).

GSDMD pores are dynamically regulated by calcium-dependent membrane lipid composition (52). Elevated intracellular calcium activates phospholipase C, which hydrolyzes phosphatidylinositol 4, 5-bisphosphate (PIP2) to generate diacylglycerol and inositol 1, 4, 5-trisphosphate. Simultaneously, calcium-activated phosphoinositide 3-kinase converts PIP2 to phosphatidylinositol 3, 4, 5-trisphosphate (PIP3). This calcium-induced shift in membrane phosphoinositide composition—specifically PIP3 accumulation at sites of GSDMD insertion—stabilizes the oligomeric pore structure and prevents pore closure, thereby maintaining permeability for sustained cytokine release (52). Since papain increases intracellular calcium levels in basophils (53), we reasoned that it would similarly affect airway epithelial cells. Indeed, papain increased intracellular calcium in airway epithelial cells, which was inhibited by blockade of the store-operated calcium channel Orai1 with BTP2 (**Figure 2D**). BTP2 also inhibited papain-induced enhancement of IC-LPS-mediated cell death and cytoplasmic IL-33 secretion (**Figures 2E, F**).

Calcium signaling could integrate diverse external signals to modulate inflammasome-mediated IL-33 secretion in airways. We tested whether altered calcium levels would affect pyroptosis of GSDMD-dependent secretion from airway epithelial cells. Supplementing culture media with calcium chloride IC LPS stimulation enhanced cell death (**Figure 2G**) and cytoplasmic IL-33 secretion (**Figures 2H, I**). While increased extracellular calcium activates NLRP3 and augments caspase-1 mediated GSDMD cleavage in macrophages (54), we observed no enhanced GSDMD cleavage with elevated calcium (**Figure 2J**). Furthermore, papain-augmented cell death did not require caspase-1 (**Supplementary Figures 2A, B**), and neither extracellular calcium nor papain augmented MAPK-dependent transcription (**Supplementary Figure 2C**). These data suggest calcium stabilizes GSDMD pores or promotes its activity rather than enhancing cleavage.

### Intracellular LPS sensor caspase-4 and lung epithelial cell IL-33 regulate type 2 immunity

Since caspase-4 (originally called caspase-11 in mouse) was previously shown to regulate adaptive type 2 lung responses to ovalbumin (55) and we demonstrated that caspase-4 regulates IL-33 secretion, a cytokine that activates multiple innate type 2 immune cells (56), we hypothesized that caspase-4 would also enhance innate type 2 lung immunity. First, we determined that mouse lung epithelial cells contain the required components to release IL-33 during non-canonical inflammasome activation. Whereas human IL-33 is expressed in bronchial epithelium and capillary endothelium (57), mouse lung IL-33 expression is highest in alveolar type 2 epithelial cells (AEC2) (58, 59). Re-analysis of previously published single cell sequencing of sorted *Il33*^RFP+^ lung cells identified AEC2s as the majority *IL33*-expressing cell type (**Supplementary Figure 2D**). We independently confirmed *Il33*^RFP^ expression predominantly in AEC2s, with lesser expression in fibroblasts and minimal expression in bronchial epithelial cells or lung macrophages under both baseline and papain-stimulated conditions (**Supplementary Figures 2E, F**). To confirm lung epithelial contribution to extracellular IL-33 during protease allergen challenge, we crossed *Il33*^flox^ mice to *Shh*^Cre^ mice to delete IL-33 from endodermally-derived tissues including the lung epithelium (“*Il33*^ΔAEC^”) but not endothelium, fibroblasts or hematopoietic cells, which are derived from mesoderm). Compared to their *Il33*^flox^ littermates, *Il33*^ΔAEC^ mice had reduced lung *Il33* RNA expression (**Supplementary Figure 2G**), less IL-33 protein in bronchoalveolar lavage, (**Supplementary Figures 2H, I**), and diminished lung eosinophilia following papain challenge (**Supplementary Figure 2J**). *Casp4* and *Gsdmd* were similarly expressed in AEC2s and alveolar macrophages (**Supplementary Figure 2K**). Thus, caspase-4 is expressed in AEC2s, which are the primary source of secreted IL-33 during papain challenge, and could feasibly serve as a mechanism for epithelial IL-33 release.

We then treated caspase-4-deficient (*Casp4*^-/-^) mice and wildtype littermates with three days of inhaled papain. IL-33 protein was reduced in broncho-alveolar lavage of *Casp4^-/-^* mice compared to wildtype mice (**Figure 2K**), despite similar *Il33* gene expression (**Supplementary Figure 2L**). Consistent with the role of IL-33 in activating ILC2s and stimulating eosinophil recruitment, *Casp4*^-/-^ mice had reduced lung eosinophils and ILC2s (**Figure 2L**). These data demonstrate that caspase-4 contributes to IL-33 secretion and type 2 immunity following allergen challenge.

### Variation in gasdermin D activation and IL-33 expression and release by LPS derived from different gram-negative species

**Figure 3:**
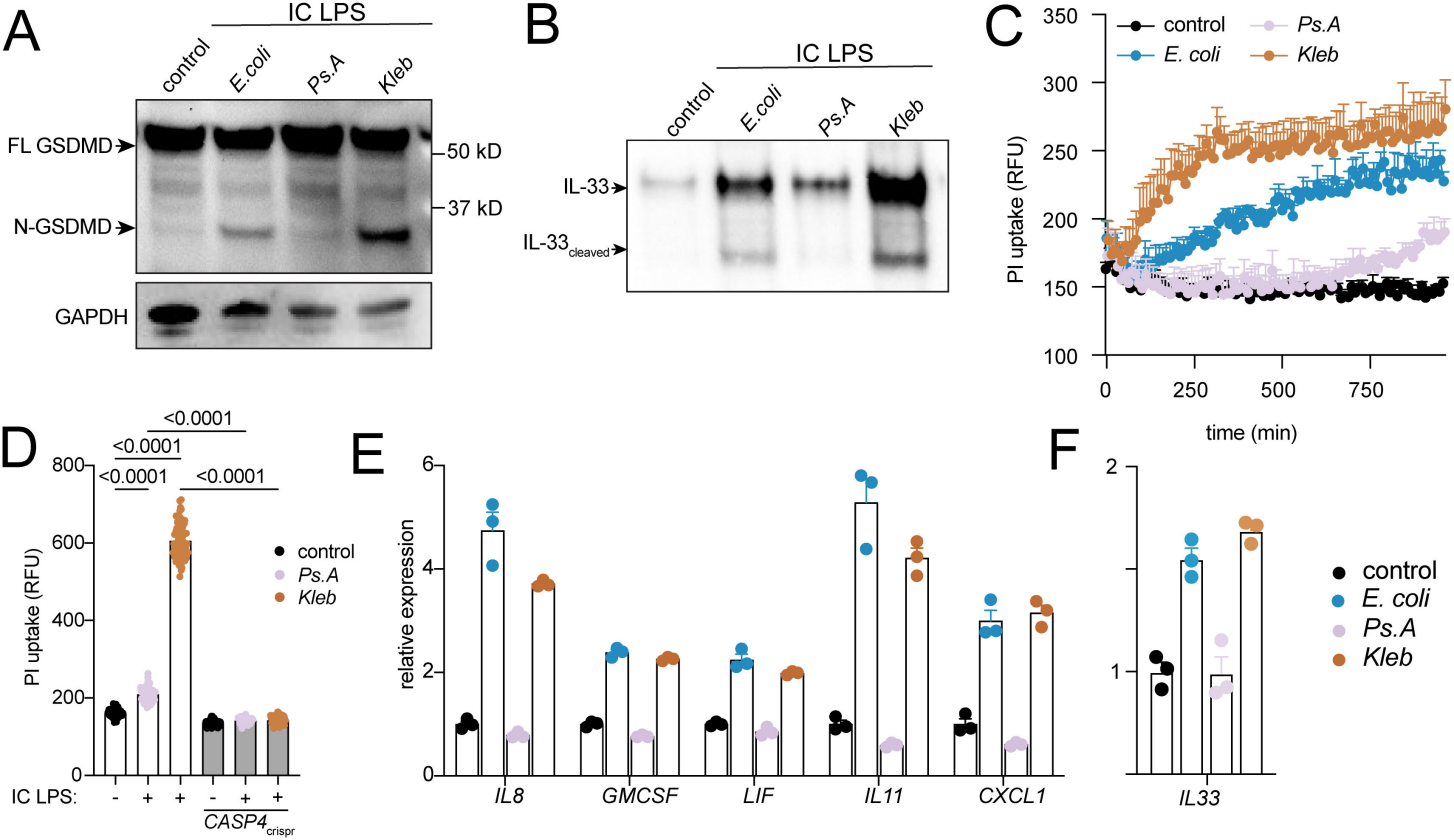
LPS from different gram-negative bacteria differ in ability to activate non-canonical inflammasome and IL-33 secretion in airway epithelia. **(A)** Western blot of GSDMD and GAPDH from cellular lysates from BEAS-2B cells treated for 3 hrs with purified LPS from three different bacterial species: *Escherichia coli* 55:B5 (*E.coli*), *Pseudomonas aeruginosa* 10.22 (*Ps.A*) and *Klebsiella pneumonia* (*Kleb*). **(B)** Western blot of IL-33 in concentrated supernatant from BEAS-2B cells treated for 16 hrs with IC LPS from different bacterial species. **(C)** Propidium iodide (PI) cell uptake over time in BEAS-2B cells lines treated with IC LPS from different bacterial species. **(D)** Propidium iodide (PI) cell uptake at 6 hours in CRISPR-BEAS-2B cells lines treated with IC LPS from different bacterial species. **(E)** MAPK and **(F)***IL33* gene expression from BEAS-2B cells treated for 4 hours with IC LPS from different bacterial species. Data points reflect biological replicates. Error bars indicate SEM. P values indicated for ordinary one-way ANOVA with Tukey correction.

We hypothesize that purified LPS from different bacterial species might differ in their ability to activate caspase-4 dependent airway epithelial IL-33 release and pyroptosis. We selected LPS from respiratory pathogens *Pseudomonas aeruginosa* and *Klebsiella pneumoniae*, and compared them to *E.coli* 055:B5, commonly used in the literature and throughout this manuscript. All three bacterial strains contain hexa-acylated lipid A but differ in their fatty acid modifications. Compared to *E.coli* LPS, *P. aeruginosa* LPS reduced, while *K. pneumoniae* LPS increased GSDMD cleavage, IL-33 secretion, and cell death (**Figures 3A–C**). Despite differences in degree of cell death induced by the different LPS species, all were *CASP4-*dependent (**Figure 3D**). MAPK signaling gene expression, including *IL33*, was similarly induced by *E.coli* and *K.pneumoniae* LPS but not by *P. aeruginosa* LPS (**Figures 3E, F**). These species-specific differences in inflammasome activation suggest that microbiome composition could potentially modulate epithelial IL-33 release and type 2 immunity.

### Gene expression of intracellular LPS sensor, caspase-4, and pyroptosis executioner, GSDMD, are increased in the airways in asthma

**Figure 4:**
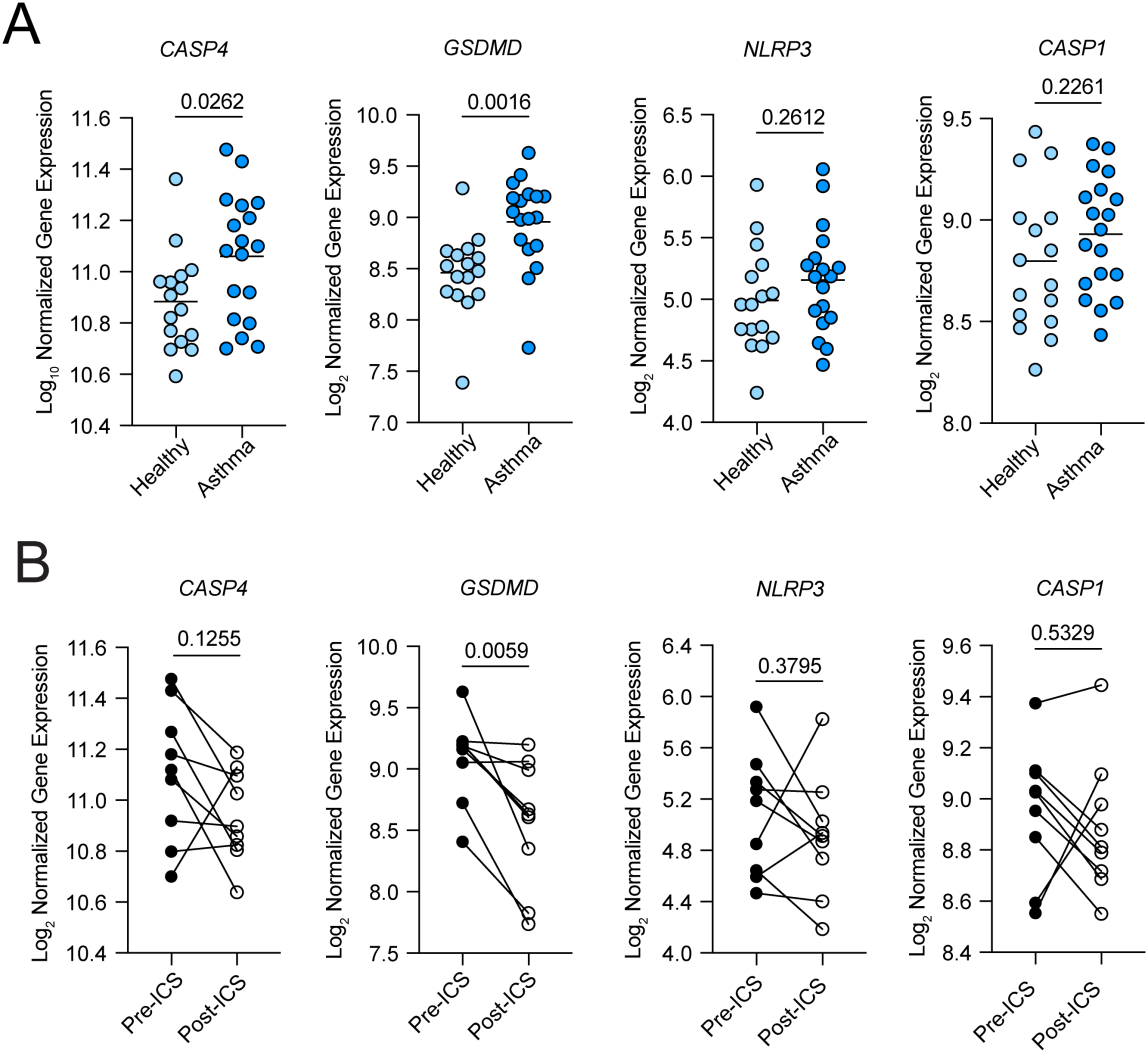
Non-canonical inflammasome components are upregulated in asthma. **(A)** Gene expression of *CASP4, GSDMD, NLRP3*, and *CASP1* in airway epithelial brushings in healthy controls and mild asthmatics. **(B)** Gene expression of *CASP4, GSDMD, NLRP3*, and *CASP1* in paired airway epithelial brushings from mild asthmatics at baseline and after 8 weeks of treatment with twice daily inhaled steroid. P values for **(A)** represent unpaired, parametric t-test. P values for **(B)** represent paired, parametric t-test. ICS, inhaled corticosteroid.

Given the importance of IL-33 in the initiation of airway type 2 inflammation and its genetic association with asthma, we investigated whether expression of caspase-4 or GSDMD might be altered in asthmatic airway epithelium. Using whole transcriptome sequencing of RNA from airway brushings obtained during research bronchoscopy, we found that *CASP4* and *GSDMD* expression were increased in mild asthmatics compared to healthy controls, while *NRLP3* and *CASP1* were not (**Figure 4A**). Moreover, inhaled corticosteroid therapy decreased *GSDMD* but not *CASP4* or *NLRP3* expression (**Figure 4B**). Bronchial brush samples are highly enriched in epithelial cells (97% epithelial cells by microscopic analysis), indicating that the observed increases in *CASP4* and *GSDMD* expression predominantly reflect epithelial cell gene expression (60). Clinical characteristics of study participants was previously published (61).

## Discussion

Allergen and microbial exposure are key factors linked to asthma development and severity; however, it is not clear how these signals are integrated at the level of key cellular sensors such as the airway epithelium. Our study provides mechanistic insight into innate immune signaling by demonstrating that intracellular LPS sensing via caspase-4 can trigger IL-33 expression and secretion as well as inflammatory MAPK activation. In cultured airway epithelial cells, intracellular LPS induces GSDMD-dependent pyroptosis, release of cytoplasmic IL-33, and MAPK signaling through the non-canonical inflammasome. These processes are amplified by the protease allergen papain through calcium signaling *in vitro*, suggesting potential convergence of microbial sensing and allergen damage pathways on shared pore-forming mechanisms. In mice, IL-33 secretion and the recruitment of ILC2s and eosinophils to the lung in response to papain are reduced in caspase-4–deficient animals. LPS from different respiratory pathogens vary in their ability to activate the non-canonical inflammasome in cultured airway epithelial cells, suggesting that microbiome composition could potentially modulate this pathway. Finally, airway brushes from asthmatic patients show elevated *GSDMD* and *CASP4* expression compared to healthy subjects, suggesting potential dysregulation of this pathway in disease.

Although the human airway is not sterile and is known to harbor a low but persistent microbial biomass, intracellular infection of airway epithelial cells by gram-negative bacteria is not a common event in healthy individuals (62, 63). Therefore, situations in which airway epithelial cells encounter intracellular LPS and which are modeled here likely represent disease-associated pathophysiologic states rather than normal respiratory homeostasis. However, several scenarios could enable epithelial access to cytosolic LPS. Intracellular pathogens including *Chlamydia pneumoniae* (64, 65), *Legionella pneumophila* (66, 67), and certain strains of *Pseudomonas* (68, 69) can invade epithelial cells, and observational studies have linked some of these organisms to exacerbations of asthma and chronic obstructive pulmonary disease (70–74). Additionally, epithelial barrier disruption caused by viral infections or protease allergens could permit LPS or bacterial access to the cytoplasm by promoting efferocytosis or trogocytosis (75–78). Outer membrane vesicles released by gram-negative bacteria can also deliver LPS into the cytosol (79–82). Future studies using models of bacterial infection or colonization will be important for establishing the importance of this mechanism in respiratory disease.

Multiple observational studies have shown an altered microbiome in patients with allergic airway diseases such as asthma and nasal polyps (9, 83–87), while acute airway infections are directly linked to exacerbations in COPD (88, 89) and asthma (90–92). In addition to the varying potential of microbial products to reach the cytosol depending on the species or pathophysiologic conditions, our finding that LPS from different bacterial strains varies in its ability to activate the non-canonical inflammasome in airway epithelial cells raises the possibility that microbial influence on asthma incidence or severity may also depend on the specific molecular moieties expressed by these pathobionts (93, 94). Whether changes in microbial community structure directly promote type 2 immunity via caspase-4–dependent IL-33 secretion remains unclear. The increased expression of the intracellular LPS sensor caspase-4 in airway epithelium from asthmatic patients supports the need to further investigate the link between host factors and microbial communities in asthma pathogenesis, including potential correlations with disease severity in larger patient cohorts.

At first glance, stimulation of type 2 immunity by an LPS sensor seems at odds with traditional models of inflammation, based largely on *in vitro* studies, which define distinct sensing inputs and effector outputs for type 1, type 2, and type 3/17 responses (95, 96). *In vivo*, however, immune responses are often mixed, and type 2 immune mediators participate in protective important roles across diverse inflammatory conditions. For instance, type 2 immunity initiated after type 1 priming may enhance neutrophil recruitment (97), drive mucus production and epithelial repair to limit bacterial dissemination (98, 99), and remodel mucus to favor symbiotic mucophilic bacteria that reduce pathobiont colonization (100, 101). Within this framing, excessive IL-33 activity and type 2 immunity in allergic disease could represent dysregulated activation of normally self-limited protective programs—or even hijacking of mutualistic pathways by pathobionts (102).

Our study also contributes to the growing understanding of how IL-33 secretion from airway epithelial cells can occur through cell death–related mechanisms. Reported pathways include unregulated necrosis leading to loss of nuclear and plasma membrane integrity (103), stress-granule formation (18), exosomal secretion (47), nuclear pore formation in response to mechanical stress (51), ripoptosome-dependent apoptosis (104), direct activation of GSDMD by protease allergen (18), and direct plasma membrane disruption by allergen-derived toxins (19). The GSDMD-dependent mechanism described here represents an additional potential route for IL-33 export, which may be particularly relevant during epithelial responses to intracellular bacteria. Notably, we did not detect secretion of full-length nuclear IL-33, suggesting that GSDMD-mediated release in our model system primarily involves cytosolic IL-33 pools that are generated either through alternative splicing (51) or nuclear export (18, 47, 51, 105). Notably, while our *in vitro* experiments demonstrate preferential release of cytoplasmic IL-33, we did not specifically identify the IL-33 species released in mice during allergen challenge. Therefore, the mechanisms that facilitate cytoplasmic localization of IL-33 and precede GSDMD-mediated secretion *in vivo* require careful future study.

Our work also suggests distinct methods of cell death in epithelium as compared to immune cells in response to the similar stimuli. While intracellular LPS leads to non-canonical inflammasome activation and pyroptotic cell death in respiratory epithelial cells just as in macrophages (24, 106), ligands that activate the canonical inflammasome in macrophages (23, 107, 108) caused apoptosis in respiratory epithelial cells. This suggests that epithelial cells and macrophages have evolved cell-type-specific inflammasome responses reflecting their distinct functions. While macrophages undergo inflammatory pyroptosis to rapidly alert the immune system, epithelial cells may favor apoptosis as a more controlled response that maintains barrier integrity during routine threats. The selective pyroptotic response of epithelial cells to intracellular LPS—signaling invasive rather than surface bacterial colonization—may represent appropriate escalation to severe threats requiring rapid IL-33 release and immune activation. This cell-type-specific tuning of inflammasome responses requires further investigation.

Our study also offers insight into how the airway epithelium integrates diverse environmental cues. In addition to the role of genetics, human observational studies show that both early life exposures to microbes and inhaled allergens influence the propensity towards developing type 2 inflammation of the airway (7, 109–115), yet the manner in which these environmental signals interact is poorly understood. Here, we elucidate one mechanism by which protease allergen can augment non-canonical inflammasome signaling initiated by intracellular LPS through activation of store operated calcium channels in cultured epithelial cells. While we used papain as a model protease allergen, this calcium-dependent potentiation mechanism is likely not papain-specific, as multiple allergens are known to mobilize intracellular calcium through various pathways, including protease-activated receptors, direct membrane disruption, and purinergic signaling (17, 116–118). Experimental validation using additional allergen types will be necessary to establish the generalizability of this calcium-dependent potentiation pathway. Whether this calcium-dependent potentiation occurs during *in vivo* allergen challenge also requires validation using pharmacological and genetic Orai1 inhibition in animal models.

In summary, we demonstrate a previously unappreciated role for caspase-4 to regulate IL-33 secretion from airway epithelial cells *in vitro* and to modulate type 2 immunity *in vivo*. While our *in vitro* studies and epithelial IL-33 deletion experiments support an important role for epithelial caspase-4, we cannot exclude contributions from immune cell caspase-4 to the observed phenotypes, and look forward to future studies in mice epithelial-specific *Casp4* deletion. Our findings suggest a mechanistic framework linking caspase-4-dependent IL-33 secretion to type 2 inflammation, with epithelial cells representing a major but not exclusive cellular source of this response. While important questions remain regarding the contexts in which airway epithelial cells encounter cytosolic LPS and the cell-type-specific contributions of caspase-4 *in vivo*, this work identifies the non-canonical inflammasome as a pathway capable of connecting microbial pattern recognition to allergic inflammation, with epithelial cells serving as important contributors to this response. The increased expression of caspase-4 and gasdermin D in asthmatic airways supports further investigation of the link between host factors and microbial communities in asthma pathogenesis, and provides a mechanistic framework for future therapeutic development.

## Materials and methods

### Mice

*Shh*^GFP-Cre^ and *Il33*^flox-GFP^ mice were obtained from Jackson Laboratories (stock numbers 005622, and 030619, respectively) and interbred to generate *Il33*^ΔAEC^ mice. *Casp4*^-/-^ (Casp4^tm1Yuan^; Strain 024698) mice were obtained from Jackson Laboratory and intercrossed to C57Bl/6J from Jackson Laboratory to obtain *Casp4*^-/-^ and wildtype littermate controls. *Il33*^RFP^ mice (Jax stock 037797) were provided by Ari Molofsky.

Mice were housed under specific pathogen-free conditions in individually ventilated cages with autoclaved bedding on 12 hr light/day cycles and *ad libitum* access to irradiated food (PicoLab Mouse Diet 20, 5058M) and autoclaved water. All animals were manipulated using standard procedures including filtered air exchange stations, chlorine-based disinfection of gloves and work surfaces within manipulations with animals. Experiments were performed on age- and sex-matched male and female mice or their littermate controls between 6–12 weeks of age.

40 μL of PBS solution (or PBS alone) was dropped by pipette onto the vocal cords of isoflurane-anesthetized mice for 3 daily doses. Tissues were analyzed on the 4^th^ day.

For bronchoalveolar lavage (BAL), 1.5mL of PBS was instilled and withdrawn via tracheal cannula, then immediately combined with protease inhibitor cocktail (Thermo, 78430) and kept on ice. Cells were removed from the lavaged fluid by gentle centrifugation at 300g at 4C. Supernatants were frozen at -80C until final analysis. Whole lung was homogenized in RIPA buffer (Thermo, 89900) with protease inhibitor (Thermo, 78430) in 2 ml tubes prefilled with 3.0 mm zirconium homogenizer beads in Bead Bug6 (Benchmark) for 60 seconds at 4350 rpm.

All experiments were approved by the UCSF IACUC and all care of animals was in accordance with institutional guidelines.

### Cell isolation & flow cytometry

Mice were perfused with PBS via the right ventricle. Single cell suspensions were obtained by digestion of the lung with 50 μg/mL LiberaseTM (Roche) and 25 μg/mL DNAse I (Roche), followed by mechanical dissociation on a GentleMACS dissociator (Miltenyi). Suspensions were filtered through a 100 μM strainer, depleted of red blood cells using PharmLyse (BD), and then passed through a 40 μM strainer prior to staining. Dead cells were gated using DAPI exclusion. Stained cells were run on a BD Fortessa flow cytometer and analyzed using FlowJo. Immune cells were gated as: ILC2s as lin–, Thy1.2+, ST2+; alveolar macrophages as Ly6g-, CD11b-, CD11c+, SiglecF+; eosinophils as CD11b+, SiglecF+, CD11c–; neutrophils were defined as CD11b+, Ly6g-hi. Structural cells were gated as: BECs as CD31-, CD45-, EpCAM+, CD24+; AEC2s as CD31-, CD45-, EpCAM+, MHCII+, and fibroblasts as CD31-, CD45-, EpCAM-, PDGFRa+.

### Immunofluorescence

Perfused mouse lungs were inflated with 2% low melt agarose in PBS and then fixed in 2% PFA overnight. Following 30% sucrose protection, lungs were embedded in OCT and frozen. 10 μM sections were affixed to Superfrost plus slides (Fisher) and stained with anti-proSPC (Millipore AB3786), anti-RFP (LSBio C340696), anti-CD45 (BD clone 30-F11) followed by secondary antibodies and DAPI for nuclear counterstain.

### IL-33 overexpressing cell lines

Plasmids containing full-length and spliced (Δ34) IL-33 open-reading frames previously described (46) were used to clone IL-33-AcGFP into pLVX-IRES-Neo (Takara, cat 632181). The vector was digested with BamHI (New England Biolabs) and the linearized plasmid was gel-purified. The following primers were used to amplify IL-33 ORF in frame with AcGFP using CloneAmp HiFi PCR Premix and the PCR product was gel-purified. FW_GAGCGGCCGCGGATCATGAAGCCTAAAATGAAGTAT TCAACCAACAAAA; RW_GAGAGGGGCGGGATCTCACT TGTACAGCTCATCCATGC.

The inserts were cloned into the linearized vector using IN-Fusion HD Cloning Plus Kit (Clontech, cat 638910). Clones were verified by capillary sequencing.

10 ug of lentiviral plasmids (pLVX-CMV-full length-IL33-AcGFP-Neo or pLVX-CMV-Δ34-IL33-AcGFP-Neo) were co-transfected into HEK-293 cells (60% confluent in 10 cm dish) with 10 μg of psPAX2 (Addgene) and pCMV-VSV-G (Addgene) using Lipofectamine in OptiMEM. Media was collected at 36 and 48 hours, filtered through a 0.45 μm filter and concentrated 10x with Lenti-C Concentrator (Clontech). Approximately 250, 000 BEAS-2B cells were cultured in a 6 well dish at 50- 60% confluence, and approximately 1.25 x10^6^ transducing units lentiviral particles (5 MOI) were applied overnight with polybrene. Cells were selected for 48 hours in 500 μg/ml G418 (Geneticin, Gibco). The highest 10% of GFP expressing cells were sorted and cultured for further experimentation. BEAS-2B cells transduced with lentivirus encoding Δ34 IL-33 splice variant are identified as “IL-33 OE” while BEAS-2B cells transduced with lentivirus encoding the full-length IL-33 transcript are identified as IL-33_NLS_ OE.

### CRISPR-Cas9 knockout cell line generation

LentiCRISPRV2 (Addgene, plasmid 52961) was linearized with BsmBI and gel purified. sgRNA oligos (**Supplementary Table 2**) were annealed using T4 PNK (New England Biolabs) and ligated into linearized plasmid using Quick T4 Ligase (New England Biolabs). Plasmid clones were verified by capillary sequencing.

10 μg of LentiCRISPRV2-sgRNA plasmids were co-transfected into HEK-293 cells (60% confluent in 10 cm dish) with 10 ug of psPAX2 (Addgene) and pCMV-VSV-G (Addgene) using Lipofectamine in OptiMEM. Media was collected at 36 and 48 hours, filtered through a 0.45 μm filter and concentrated 10x with Lenti-C Concentrator (Clontech). Viral titer determined by LentiX p24 Rapid Titer kit. Approximately 250, 000 BEAS-2B cells were cultured in a 6 well dish at 50- 60% confluence, and approximately 1.25 x10^6^ transducing units lentiviral particles (5 MOI) were applied overnight with polybrene. After 48 hours, cells were selected with 2 μg/ml puromycin (Invivogen) for 48hours. Knock down was verified by Western blot (**Supplementary Figure 1B**).

### Cell culture and stimulation

IL-33- overexpressing BEAS-2B airway epithelial cells were cultured in RPMI with 10% fetal calf serum. Cells were treated with intracellular LPS, 2 μg/ml LPS (*E. coli* O55:B5, ultrapure, Invivogen; *Pseudomonas aeruginosa 10* L9143, Sigma; *Klebsiella pneumoniae*, Sigma L4268) with 0.45% v/v Fugene HD (Promega), dA:dT (Invivogen) 1 μg/ml with 0.45% v/v Fugene HD, or Nigericin 5 μM (Sigma-Aldrich) in OptiMEM (Thermo). Papain (1 unit/ml, Worthington, LS003124). BTP2 (10 μM, Cayman Chemical, YM-58483, cat 13246), calcium chloride (1 mM) or Trametinib (10 μM MedChemExpress, cat HY-10999) were added as indicated. Intracellular calcium flux was measured using Fluo-8 AM fluorescence measured at 490/520nm.

Primary airway epithelial cells were harvested from cadaver tracheas obtained from Donor Network West (119). Primary human basal airway epithelial cells from tracheal digests were expanded at 37 °C in media (67.5% DMEM-F, 25% Ham’s F-12, 7.5% FBS, 1.5 mM l-glutamine, 25 ng/mL hydrocortisone, 12 5 ng/mL EGF, 8.6 ng/mL cholera toxin, 24 μg/mL Adenine, 0.1% insulin, 75 U/mL pen/strep) supplemented with ROCK1 Inhibitor (RI, 16 μg/mL), and antibiotics (1.25 μg/mL amphotericin B, 2 μg/mL fluconazole, 50 μg/mL gentamicin. Primary cells (10, 000/ul) were exposed to electroporation or sham in the presence of LPS (2 μg/ml, ultrapure, Invivogen) or dA:dT (2 μg/ml, Invivogen) using the Neon Transfection System (Invitrogen) (1 pulse, 1200V, 40 ms). Following electroporation cells were cultured for 16 hours in 96 well plate prior to harvest.

Human alveolar macrophages were isolated from human donor lungs were obtained from Donor Network West. A bronchoalveolar lavage (BAL) was done in the left upper lobe using PBS. BAL cells were centrifuged 300g 10min, resuspended in RPMI + 10%FCS and cultured at 37 degrees for 2.5 hours. Media was removed and cells were lysed in RLT buffer for RNA analysis.

### Cell death assays

Propidium iodide uptake (P4864, Sigma) was added at a concentration 2 ug/ml and 533 excitation/617 emission fluorescence was recorded every 10 minutes for 16 hours at 37 degrees (Synergy H1, Biotek). CyQuant Cytotoxicity Assay Kit (Thermo) was used to measure G6PD release from BEAS-2B cell conditioned media. Caspase-Glo 3/7 Assay Kit (Promega, G8090) was used to measure caspase 3/7 activity in BEAS-2B cell culture lysates.

### IL-33 activity assay

HEK-Blue IL-33 Cells (Invivogen cat hkb-hil33) were cultured according to manufacturer’s instructions. 50 μL of cell culture supernatant cleared of cell debris was incubated with 42, 000 reporter cells overnight in 96 well flat bottom dish. 20 μL of HEK-blue conditioned media was then combined with 180ul of QUANTI-Blue (Invivogen, pre-qbs) for 4 hours at 37 degrees and absorption at 620 nm was read on a spectrophotometer.

### RNA expression analysis

Tissue was homogenized in RLT buffer (Qiagen) using 2 ml tubes prefilled with 3.0 mmzirconium homogenizer beads in Bead Bug6 (Benchmark) for 60 seconds at 4350 rpm. Sorted cells or cell lines were collected directly in RLT buffer. RNA was isolated using the RNeasy mini kit (Qiagen), and then reverse transcribed using Vilo Superscript III (Invitrogen). Quantitative PCR using Taqman PCR mix (Applied Biosystems) was performed using primers specified in **Supplementary Table 1**) on QSpro Thermocycler (Applied Biosystems). qPCR gene expression for human target genes was normalized to the geometric mean of three housekeeping genes: *RPL13A, PPIA, EEF1A1* as previously described (120). qPCR gene expression for mouse target genes was normalized to *Gapdh*. Bronchial brush gene expression from healthy controls and asthmatics before and after inhaled corticosteroids was obtained from publicly available dataset GSE164119. Single cell representation of sorted *Il33*^RFP^+ lung cells was obtained from published and publicly available data GSE125492 (121). R and the R package Seurat were used to re-visualize and assign identities to cells according to the published workflow (121, 122).

### Protein analysis

Conditioned media cleared of cellular debris was assayed for GFP using an ELISA kit from Cell Biolabs, Inc (cat AKR-121). Cultured cell lines were lysed in RIPA buffer with protease inhibitor by scraping. Lysate was cleared of debris by centrifugation at 20, 000 g for 20 minutes. Supernatant was assayed for total protein using microBCA kit (Thermo, 23235). Conditioned media from cultured cells was immediately combined with protease inhibitor cocktail (Thermo, 78430), cleared of cell debris by centrifugation at 300 g for 10 minutes at 4 degrees and then used in downstream assays. Protein was precipitated from cell culture conditioned media for the purpose of Western blot analysis by combining with 4x volume of ice cold acetone and incubating at -20 for 1–2 hours followed by centrifugation at 15, 000 g for 10 minutes at 4 degrees. Protein pellet was resuspended in Laemlli buffer and heated to 100C for 10 minutes. Primary human airway epithelial cells were lysed in Laemlli buffer prior to heating at 100C for 10 minutes.

### Western blots

20 μg of protein lysates from lung homogenates and cell culture or 35 ul of lavage fluid were combined with 4x Laemelli buffer (Thermo) and heated to 100 degrees for 10 minutes. Samples were loaded on NuPage 4-12% Bis-Tris gels (Thermo, NP0335). Proteins were transferred to PVDF membrane using the iBlot2 gel transfer system (Thermo). Membranes were incubated in 1% BSA (biotinylated primary antibody) or 5% nonfat milk (non-biotinylated antibody) for 1 hour at room temperature. Membranes were incubated overnight at 4 degrees with indicated antibody, washed with 1% TBS-Tween, probed with secondary antibody (Cell Signaling, goat anti-rabbit HRP, 1:2000) or strep-avidin HRP (Cell Signaling, 1:2000), washed again, and exposed with ProSignal Femto ECL (Prometheus). Primary antibodies used on mouse lavage: biotinylated anti-IL33 (R&D, AF3625, 1:1000). Primary antibodies used in human cell culture or conditioned media: biotinylated anti-IL33 Nessy-1 (Enzo, ALX-840-C100, 1:1000), anti-GSDMD (Cell signaling E8G3F, 1:1000), GAPDH antibody (Proteintech, 10494-1-AP, 1:5000), Caspase-1 (Cell Signaling, 2225S, 1:500) and Caspase-4 (Cell Signaling, 4450, 1:500).

## Data Availability

The datasets presented in this study can be found in online repositories. The names of the repository/repositories and accession number(s) can be found in the article/**Supplementary Material**.

## Supplementary material

Supplementary Figure 1 **(A)** Gene expression of *NLRP3, CASP1, CASP4*, and *GSDMD* in cultured primary human alveolar macrophages, cultured human primary airway epithelial cells, and BEAS-2B airway epithelial cell line. **(B)** Western blot of Caspase 1, Caspase 4, GSDMD, and GAPDH on lysates from CRISPR-Cas9 genetically modified BEAS-2B cell lines (scramble _crispr_, CASP1_crispr_, CASP4 _crispr_, GSDMD _crispr_) **(C)** Western blot of IL-33 in concentrated supernatant from nuclear IL-33 (IL-33_NLS_ OE) over-expressing BEAS-2B cell following 16 hour treatment with IC LPS, dA:dT, or nigericin. **(D)** IL-33 activity measured by secreted embryonic alkaline phosphatase from HEK-Blue-IL33 cells treated with media from IL-33_NLS_OE or IL-33OE BEAS-2B cells treated with IC LPS, dA:dT or nigericin for 16 hours. **(E)** Propidium iodide (PI) cell uptake over time in BEAS-2B cells treated with intracellular LPS (IC LPS), AIM2 activator dA:dT, or NLRP3 activator nigericin. **(F)** Caspase-3/7 activity measured in cell lysate following 16 hour stimulation with IC LPS, dA:dT, or nigericin. **(G)** PI uptake over time in CRISPR-BEAS-2B cells treated with IC LPS or dA:dT **(H)** Glucose 6-phosphade dehydrogenase (G6PD) activity measured in media from indicated CRISPR-BEAS-2B cells treated with IC LPS for 16 hours. **(I)** Western blot of IL-33 in concentrated supernatant from cytoplasmic IL-33 (IL-33 OE) and scramble or CRISPR-BEAS-2B cells following 16 hour treatment with IC LPS. Data points reflect biological replicates. Error bars indicate mean and SEM. P values two-way ANOVA with Sidek correction **(A, H)** and Tukey correction **(F)**.

Supplementary Figure 2 **(A)** PI uptake at 6 hours in *CASP1* Crispr-Cas9 genetically modified BEAS-2B cells treated with IC LPS and papain. **(B)** PI uptake over time in *CASP1* Crispr-Cas9 genetically modified BEAS-2B cells treated with IC LPS and calcium chloride. P value represents one **(A)** and two-way **(B)** ANOVA with Sidak comparison. (**C**) MAPK gene expression from BEAS-2B cells treated for 4 hours with IC LPS with or without calcium chloride, papain or MAPKi trametinib. **(D)** Composition of RFP^+^ cells from lungs of *Il33*^RFP^mice from published single cell sequencing. **(E)** Mean fluorescence intensity of *Il33*^RFP^ in cells isolated from the lung of *Il33*^RFP^ mice after 3 days of intratracheal PBS or papain. **(F)** Immunofluorescent microscopy of lung from *Il33*^RFP^ mice probed with SPC (green) or CD45 (white) antibody and counterstained with DAPI (blue). Large scale bar 100 μm; inset scale bar 20 μm **(G)***Il33* gene expression in lung homogenate from *Il33*^flox^ (Il33^fl/fl)^ and *Shh*^cre+^*Il33*^flox^ mice (*Il33*^ΔAEC^). **(H)** Western blot of BAL showing IL-33 protein wildtype mice treated with 3 days of intratracheal PBS or papain. **(I)** Western blot of BAL showing IL-33 protein in *Il33*^fl/fl^ and *Il33*^ΔAEC^ mice treated with 3 days of intratracheal papain. **(J)** Representative flow cytometry of CD45^+^, CD11c^-^, Siglec-F^+^ eosinophils from lungs of *Il33*^fl/fl^ and *Il33*^ΔAEC^ mice after 3 days of intratracheal papain, with quantification. **(K)** Gene expression analysis of sorted AEC2s and AMPs showing relative expression of *Il33* and inflammasome components (*Nlrp3, Casp1, Casp4, Gsdmd*). **(L)** Relative expression of *Il33* mRNA in whole WT and *Casp4^-/-^ mouse lung.* BEC, bronchial epithelial cell. AEC2, type 2 alveolar epithelial cells. Eos, eosinophils. AMP, alveolar macrophage. Fibro, fibroblast. RFP, red fluorescent protein. SPC, surfactant protein C. BAL, bronchoalveolar lavage. Data points reflect biological replicates. Error bars indicate SEM. P values indicated for student’s T-test **(G, J, K, L)** ordinary one-way ANOVA with Tukey correction **(A, B)**.

Supplementary Figure 3 Full length and Uncropped Western Blots.

Supplementary Table 1 Quantitative PCR Primers.

Supplementary Table 2 Single guide RNA Oligos.
