# Supplementary figures and images for: Microbial sensing through the non-canonical inflammasome modulates airway type 2 immunity
Source: Front Immunol. 2026 Mar 16;17:1784561. doi: 10.3389/fimmu.2026.1784561 (PMC13033652; doi:10.3389/fimmu.2026.1784561)

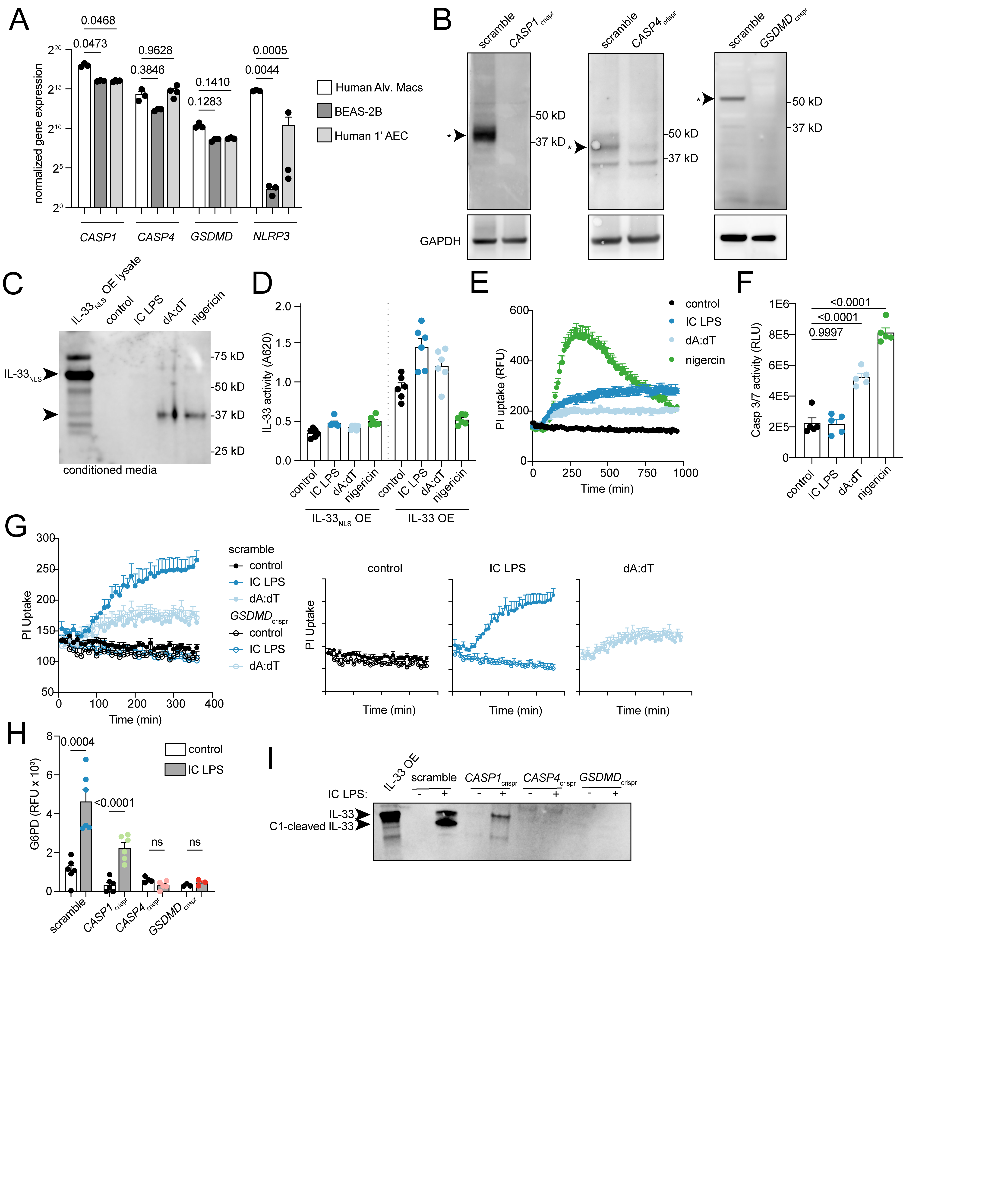

Supplement: Supplementary Figure 1 — (A) Gene expression of NLRP3, CASP1, CASP4, and GSDMD in cultured primary human alveolar macrophages, cultured human primary airway epithelial cells, and BEAS-2B airway epithelial cell line. (B) Western blot of Caspase 1, Caspase 4, GSDMD, and GAPDH on lysates from CRISPR-Cas9 genetically modified BEAS-2B cell lines (scramble crispr, CASP1crispr, CASP4 crispr, GSDMD crispr) (C) Western blot of IL-33 in concentrated supernatant from nuclear IL-33 (IL-33NLS OE) over-expressing BEAS-2B cell following 16 hour treatment with IC LPS, dA:dT, or nigericin. (D) IL-33 activity measured by secreted embryonic alkaline phosphatase from HEK-Blue-IL33 cells treated with media from IL-33NLSOE or IL-33OE BEAS-2B cells treated with IC LPS, dA:dT or nigericin for 16 hours. (E) Propidium iodide (PI) cell uptake over time in BEAS-2B cells treated with intracellular LPS (IC LPS), AIM2 activator dA:dT, or NLRP3 activator nigericin. (F) Caspase-3/7 activity measured in cell lysate following 16 hour stimulation with IC LPS, dA:dT, or nigericin. (G) PI uptake over time in CRISPR-BEAS-2B cells treated with IC LPS or dA:dT (H) Glucose 6-phosphade dehydrogenase (G6PD) activity measured in media from indicated CRISPR-BEAS-2B cells treated with IC LPS for 16 hours. (I) Western blot of IL-33 in concentrated supernatant from cytoplasmic IL-33 (IL-33 OE) and scramble or CRISPR-BEAS-2B cells following 16 hour treatment with IC LPS. Data points reflect biological replicates. Error bars indicate mean and SEM. P values two-way ANOVA with Sidek correction (A, H) and Tukey correction (F). [file Image1.tif]

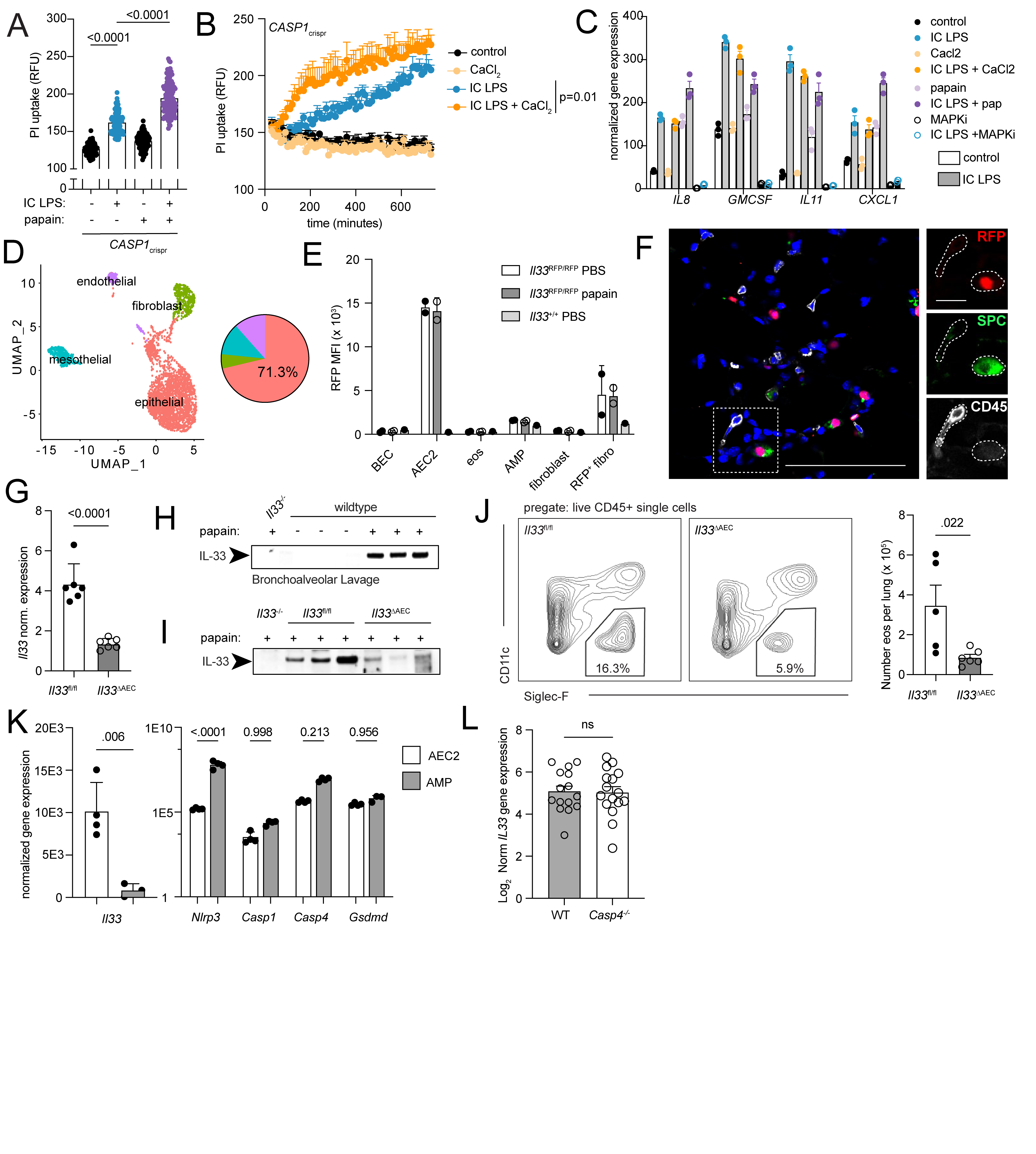

Supplement: Supplementary Figure 2 — (A) PI uptake at 6 hours in CASP1 Crispr-Cas9 genetically modified BEAS-2B cells treated with IC LPS and papain. (B) PI uptake over time in CASP1 Crispr-Cas9 genetically modified BEAS-2B cells treated with IC LPS and calcium chloride. P value represents one (A) and two-way (B) ANOVA with Sidak comparison. (C) MAPK gene expression from BEAS-2B cells treated for 4 hours with IC LPS with or without calcium chloride, papain or MAPKi trametinib. (D) Composition of RFP+ cells from lungs of Il33RFPmice from published single cell sequencing. (E) Mean fluorescence intensity of Il33RFP in cells isolated from the lung of Il33RFP mice after 3 days of intratracheal PBS or papain. (F) Immunofluorescent microscopy of lung from Il33RFP mice probed with SPC (green) or CD45 (white) antibody and counterstained with DAPI (blue). Large scale bar 100 μm; inset scale bar 20 μm (G)Il33 gene expression in lung homogenate from Il33flox (Il33fl/fl) and Shhcre+Il33flox mice (Il33ΔAEC). (H) Western blot of BAL showing IL-33 protein wildtype mice treated with 3 days of intratracheal PBS or papain. (I) Western blot of BAL showing IL-33 protein in Il33fl/fl and Il33ΔAEC mice treated with 3 days of intratracheal papain. (J) Representative flow cytometry of CD45+, CD11c-, Siglec-F+ eosinophils from lungs of Il33fl/fl and Il33ΔAEC mice after 3 days of intratracheal papain, with quantification. (K) Gene expression analysis of sorted AEC2s and AMPs showing relative expression of Il33 and inflammasome components (Nlrp3, Casp1, Casp4, Gsdmd). (L) Relative expression of Il33 mRNA in whole WT and Casp4-/- mouse lung. BEC, bronchial epithelial cell. AEC2, type 2 alveolar epithelial cells. Eos, eosinophils. AMP, alveolar macrophage. Fibro, fibroblast. RFP, red fluorescent protein. SPC, surfactant protein C. BAL, bronchoalveolar lavage. Data points reflect biological replicates. Error bars indicate SEM. P values indicated for student’s T-test (G, J, K, L) ordinary one-way ANOVA with Tukey correction (A, B). [file Image2.tif]

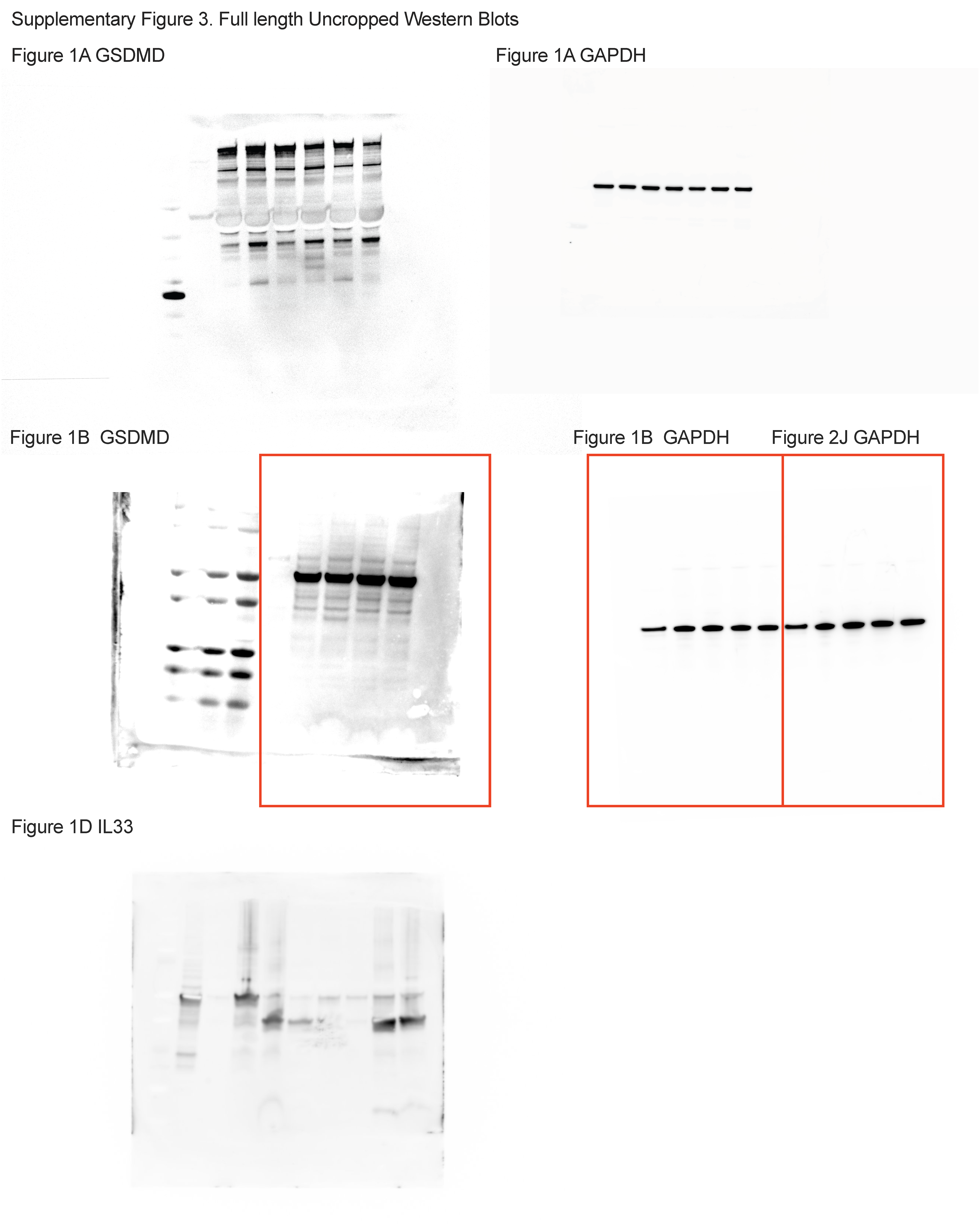

Supplement: Supplementary Figure 3 — Full length and Uncropped Western Blots. [file Image3.tiff]
