# Supplementary material for: Microbial sensing through the non-canonical inflammasome modulates airway type 2 immunity
Source: Front Immunol. 2026 Mar 16;17:1784561. doi: 10.3389/fimmu.2026.1784561 (PMC13033652; doi:10.3389/fimmu.2026.1784561)
Supplement: Supplementary Table 1 — Quantitative PCR Primers. [file DataSheet1.pdf]

Mouse		Human	
m_Gapdh FW	TGTGTCCGTCGTGGATCTGA	h_CASP1_FW	TCACTGCTTCGGACATGACTACA
m_Gapdh RW	CCTGCTTCACCACCTTCTTGAT	h_CASP1_RW	GGAACGTGCTGTCAGAGGTCTT
m_Gapdh_Probe	CCGCCTGGAGAAACCTGCCAAGTATG	h_CASP1_Probe	CATTTGCACACCGCCCAGAGCA
m_Il33_FW	TCGCTGATTTCCAAGGAGCTA	h_NLRP3_FW	GGGCAGCCTTCAGTCTGATTC
m_Il33_RW	AAAGCAAGCAGCCGAGACAT	h_NLRP3_RW	CCACTCTCCATCTGCTGTTTCAG
m_Il33_Probe	CATCCAAGCAGATGCATGCATCCA	h_NLRP3_Probe	AACGAGGTCCTCTTCACCATGTGC
m_Casp1_FW	GCCTGCCCAGAGCACAAG	h_CASP4_FW	TCATGGCAGACTCTATGCAAGAG
m_Casp1_RW	ATATTCCCTCCTGGATACCATGAG	h_CASP4_RW	GGTGGTCCAGCCTCCATATTC
m_Casp1_Probe	TCTGACAGTACTTTCCTTGTATTCATG	h_CASP4_Probe	AAGCAACGTATGGCAGGACAAATGC
m_Ppia_FW	AGGATGAGAACTTCATCCTAAAGCA	h_GSDMD_FW	CCAGCAGGGCAGAGTGTTTG
m_Ppia_RW	CGTTTGTGTTTGGTCCAGCAT	h_GSDMD_RW	CCTTCGTATGCCACCTTTATTGTG
m_Ppia_Probe	CCATGGACAAGATGCCAGGACCTG	h_GSDMD_Probe	CCCACCAGCTGCTAGCCCTAGG
m_Eef1a_FW	CAAAGCTGACCCGCCTCA	h_PPIA_FW	ACGGGTCCTGGCATCTTGT
m_Eef1a_RW	CCCAGGGTGGTTCAGGATG	h_PPIA_RW	GCAGATGAAAAACTGGGAACCA
m_Eef1a_Probe	AGGCTGCCCAGTTCACCTCTCAGGTT	h_PPIA_Probe	ATGGCAAATGCTGGACCCAACACA
m_Gsdmd_FW	CTATGCCTCCCTGTTCCTATTG	h_EEF1A1_FW	CTGAACCATCCAGGCCAAAT
m_Gsdmd_RW	TGAGTATGGTTCTTGGCTTCC	h_EEF1A1_RW	GCCGTGTGGCAATCCAAT
m_Gsdmd_Probe	CCTCCCTTCCCACAACATCTCCAT	h_EEF1A1_Probe	AGCGCCGGCTATGCCCCTG
m_Casp4_FW	GCTTGCTCTTGTCATCTCTTTG	h_RPL13A_FW	TATGCTGCCCCACAAAACC
m_Casp4_RW	GTTGCTTTGTTCTCAGTTGCC	h_RPL13A_RW	CAGAGCGGCCTGGCCTCGCT
m_Casp4_Probe	TGTCAAGGTTGCCCGATCAATGGT	h_RPL13A_Probe	TGCCGTCAAACACCTTGAGA
m_Nlrp3_FW	CAGATTGCTGTGTGTGGGACTG	h_IL8_FW	ACTCCAAACCTTTCCACCCC
m_Nlrp3_RW	CTGGTCAGAGAATGGTTGGAGC	h_IL8_RW	CATCTTCACTGATTCTTGGATACCAC
m_Nlrp3_Probe	CTAAATCGCATCGGAACCAAAGGAGC	h_IL8_Probe	AAGCTTTCTGATGGAAGAGAGCTCTGTC
		h_GMCSF_FW	CCTGAAGGACTTTCTGCTTGTCA
		h_GMCSF_RW	CTCATCTGGCCGGTCTCACT
		h_GMCSF_Probe	CCCCTTTGACTGCTGGGAGCCAG
		h_IL11_FW	CTTTGGGAGGACGAAGCGA
		h_IL11_RW	CACAGCATGCAGTGGTTTTGTA
		h_IL11_Probe	TCGCAGGCCGGTCTCAAACTCTTG
		h_CXCL1_FW	TTTCTGAGGAGCCTGCAACA
		h_CXCL1_RW	TCTCATTGGCCATTTGCTTG
		h_CXCL1_Probe	CCGCCAGCCTCTATCACAGTGGC
		h_IL33_FW	ATGGCCTACCATCCCTTCTG
		h_IL33_RW	AGCAACTTTGCCCAATGTGA
		h_IL33_Probe	ACCCTGGCTTCCAGGGACCTATGTCTT
