# Supplementary material for: Microbial sensing through the non-canonical inflammasome modulates airway type 2 immunity
Source: Front Immunol. 2026 Mar 16;17:1784561. doi: 10.3389/fimmu.2026.1784561 (PMC13033652; doi:10.3389/fimmu.2026.1784561)
Supplement: Supplementary Table 2 — Single guide RNA Oligos. [file DataSheet2.pdf]

| sgRNA Oligos | |
|---|---|
| | |
| scramble_FW | CAC CGG CAC TAC CAG AGC TAA CTC A |
| scramble_RW | AAA CTG AGT TAG CTC TGG TAG TGC C |
| | |
| CASP1_FW | TAATGAGAGCAAGACGTGTG |
| CASP1_RW | CACACGTCTTGCTCTCATTA |
| | |
| CASP4_FW | TGCTGTTTACAAGACCCACG |
| CASP4_RW | CGTGGGTCTTGTAAACAGCA |
| | |
| GSDMD_FW | ACGCGCACCCACAAGCGGGA |
| GSDMD_RW | TCCCGCTTGTGGGTGCGCGT |
